# The concentrations of immunoglobulins in bovine colostrum determined by the gold standard method are genetically correlated with their near-infrared prediction

**DOI:** 10.1186/s12711-021-00681-8

**Published:** 2021-11-10

**Authors:** Angela Costa, Marco Franzoi, Giulio Visentin, Arianna Goi, Massimo De Marchi, Mauro Penasa

**Affiliations:** 1 Department of Agronomy, Food, Natural resources, Animals and Environment, University of Padova, Legnaro, PD Italy; 2 Department of Veterinary Medical Sciences, University of Bologna, Ozzano dell’Emilia, BO Italy

## Abstract

**Background:**

The quality of colostrum administered to calves is based on its concentration in immunoglobulins G (IgG, g/L). Immunoglobulins A (IgA) and M (IgM) are also present but at a lower level. The gold standard reference analysis for these traits, radial immunodiffusion, is time-consuming and expensive. In order to define breeding strategies that are aimed at improving colostrum quality in dairy cattle, a large amount of data is needed, and the use of indicator traits would be beneficial. In the study presented here, we explored the heritabilities of reference (radial immunodiffusion) and near infrared-predicted IgG, IgA, and IgM concentrations and estimated their genetic correlations. First, the colostrum of 765 Holstein cows from nine herds was sampled to perform a reference analysis and the near-infrared spectra (400–2500 nm) were stored. We used a calibration set (28% of the initial samples) that was representative of the herds and cow parity orders to develop prediction equations for IgG, IgA, and IgM concentrations. Finally, these traits were predicted in the validation set (72% of the initial samples) to estimate genetic parameters for the predictions. Genetic correlations between reference and predicted values of each trait were estimated through bivariate linear animal models.

**Results:**

The three near-infrared-predicted immunoglobulin fractions were genetically correlated with their reference value. In particular, the reference and predicted IgG concentrations were strongly correlated at both the genetic (0.854 ± 0.314) and phenotypic level (0.767 ± 0.019). Weaker associations were observed for IgA and IgM concentrations, which were predicted with lower accuracy compared to IgG. Simulation analyses suggested that improving colostrum quality by selective breeding in Holstein cattle based on near-infrared predicted colostrum immunoglobulins concentrations is feasible. In addition, less than 10 mL of colostrum are needed for spectra acquisition and thus implementation of such analyses is possible in the near future.

**Conclusions:**

The concentrations of colostrum immunoglobulins can be predicted from near-infrared spectra and the genetic correlation between the reference and the predicted traits is positive and favourable, in spite of the large standard errors of the estimates. Near-infrared spectroscopy can be exploited in selective breeding of dairy cattle to improve colostral immunoglobulins concentration.

## Background

The cotyledonary synepitheliochorial placenta of female cattle does not allow the direct transfer of immunoglobulins (Ig) from the dam to the foetus. Therefore, compared to other mammals, the quality and volume of colostrum administered to new-born calves are even more important. Acquisition of antibodies in calves occurs through a rapid and appropriate intake of good quality colostrum after birth, i.e., colostrum with a concentration of immunoglobulins G (IgG) greater than 50 g/L [1, 2]. In general, the ‘3Q’ rule (quickness, quality, and quantity) must be followed so that the optimal administration provides more than four litres of good quality colostrum during the first 12 h of life [1, 2]. A fourth ‘Q’, standing for quota, may be added; in fact, the presence of a farm colostrum bank becomes important when, for some reasons, a cow does not produce enough colostrum for her calf. Immunoglobulins A (IgA) and M (IgM) are also present in the colostrum of cows, but at lower concentrations compared to that of IgG [2, 3]. In calves, a failure of the passive transfer of immunity, i.e. of Ig, has negative consequences on survival rate, health, growth, and performance.

Direct determination of the concentration of antibodies in the colostrum relies on gold standard methods such as the radial immunodiffusion (RID), which provides very accurate and repeatable results. However, such methods are expensive and time-consuming, which hampers the quantification of these traits on a large scale. In fact, RID analysis requires expert and trained personnel, a 24-h incubation until the endpoint, and costs around 100 US $ for 20 samples. Therefore, RID is not suitable for a rapid, easy, and low-cost collection of data in cattle.

On the one hand, BRIX refractometers are commonly used to assess colostrum quality on farms since the correlation between refractive index and IgG concentration is moderate to strong, making BRIX a good screening parameter for management purposes [4, 5]. On the other hand, infrared spectroscopy is known to be a rapid and cost-effective technique for the analysis of milk and dairy products and its effectiveness in predicting several difficult-to-measure traits in dairy cattle is well documented [6, 7]. To our knowledge only a couple of studies have discussed the application of near-infrared spectroscopy (NIRS) on bovine colostrum to determine its gross composition and IgG concentration [8, 9]. In both cases, the number of samples analysed was relatively small and genetic investigations were not carried out. The collection of colostrum spectra would allow to develop prediction equations for several traits of interest, not only for farmers (e.g., IgG concentration), but also for food and pharmaceutical industries, which use bovine colostrum as an ingredient. Currently, NIRS devices are routinely used in both the food and pharmaceutical fields [10, 11], but no models have been developed for the colostral content of antibodies, vitamins, fatty acids, total protein, lactose, and fat.

The aims of the current study were to (i) develop NIRS prediction models for IgG, IgA, and IgM concentrations in the colostrum of Holstein cows, (ii) estimate the heritability and genetic correlations between measured and NIRS-predicted concentrations of Ig, and (iii) simulate the response to selection on colostrum quality if NIRS predictions are used as a proxy. Finally, in order to evaluate how the size of the training set affects the genetic parameters, different prediction models were developed for IgG, which is the predominant colostral immunoglobulin fraction, by reducing the number of samples included in the training set.

## Methods

### Sampling

Nine commercial Holstein farms in Northern Italy with herd size ranging from 60 to 190 lactating cows were enrolled in this study. Vaccination before calving against rotavirus, coronavirus, or *E. coli* was not performed on such farms and all the cows were fed a maize-based total mixed ration, housed in free stall barns, and milked twice-a-day. Colostrum samples were collected from spring 2019 to spring 2020, covering all the calving seasons.

A detailed sampling protocol was set up using information available from the existing literature and was then provided to each farmer to explain the objectives and the methodology of the trial. According to the protocol, each calf must be separated from the dam immediately after birth and was not allowed to suckle. Only the first colostrum of supervised calvings collected within 6 h postpartum was considered. Plastic sterile tubes (120 mL) without preservative were provided and used for sample collection. Farmers were in charge of colostrum sampling and annotation of the cow ID and calving date on the tube. Immediately after collection, colostrum samples were frozen and stored at −20 °C. Periodically, colostrum samples were retrieved from the farms, transferred to the laboratory of the Department of Agronomy, Food, Natural resources, Animals and Environment of the University of Padova (Legnaro, Italy), and stored frozen until the day of analysis. In total, 765 samples of purebred Holsteins (1 sample per cow) were collected.

### Reference analysis

Samples were thawed at 4 °C in a water bath overnight and were subsequently inverted for homogenization. The ‘Bovine IgG RID Kit’, ‘Bovine IgA RID Kit’, and ‘Bovine IgM RID Kit’ were purchased in advance from Triple J Farms (Bellingham, WA, US) and stored at 4 °C. Each kit consisted of one plate (24 wells) and three reference sera. In order to fall within the detection range of the assay, each colostrum sample was divided into aliquots which were diluted in pure deionized water, i.e., 1:5 (v/v) for IgG and 1:3 (v/v) for IgA and IgM. Subsequently, 5 μL of diluted colostrum was injected in each RID plate well. After incubation at 20 °C for 24 h, plates were scanned at high resolution and the diameter of the precipitated rings was measured by using the software ImageJ [12].

Dilution, RID analyses, and scans were performed by the same operator, while each precipitated ring was measured in duplicate and the average value was taken as the final diameter (mm). For each sample, the diameter was used to derive the concentration (g/L) of the target component (IgG, IgA, or IgM) using the standard equation that has been developed specifically for each single plate based on known concentrations and diameters of the three reference sera. Concentrations of the reference sera provided by the manufacturer were 1.80, 14.72, 28.03 g/L for IgG, 0.53, 1.94, 3.87 g/L for IgA, and 0.62, 2.00, and 3.81 g/L for IgM. The data from the RID analyses were considered inconsistent and set to missing values when (i) no signal was detected, (ii) the precipitation ring was too weak for a correct measurement of the precipitation diameter, (iii) precipitation rings had an elliptical shape, and (iv) concentration values were outside of the specified kit range. The final number of samples each with at least one quantified Ig fraction was 698.

The repeatability of RID analysis was determined prior to the beginning of colostrum sampling with a preliminary test to define the accuracy of the method. In particular, the intra-assay coefficient of variation (${\mathrm{CV}}_{RID}$, %) of samples tested in quintuplicate by a single operator was calculated for IgG, IgA, and IgM, respectively. Briefly, four colostrum samples were diluted in pure water as previously described and inserted in five wells (quintuplicate) of each of the three plates (IgG, IgA, or IgM). In each plate, the three reference sera were injected. After incubation at 20 °C for 24 h, the concentration of the target component was derived as described previously. Separately for IgG, IgA, and IgM plate, the ${\mathrm{CV}}_{RID}$ was calculated as the average of the individual CV of the four samples measured in quintuplicate, as:

$${\mathrm{CV}}_{RID}=\frac{\left[\left({\mathrm{s}}_{ 1}/{\overline{\mathrm{x}} }_{ 1}\right)+ \left({\mathrm{s}}_{2}/{\overline{\mathrm{x}} }_{ 2}\right)+\left({\mathrm{s}}_{ 3}/{\overline{\mathrm{x}} }_{ 3}\right)+ \left({\mathrm{s}}_{ 4}/{\overline{\mathrm{x}} }_{4}\right)\right]}{4}\cdot 100,$$

where ${\overline{\mathrm{x}} }_{\mathrm{ n}}$ and ${\mathrm{s}}_{\mathrm{n}}$ are the mean and the standard deviation (SD), respectively, of the five concentrations available for the same sample. The intra-assay ${\mathrm{CV}}_{RID}$ was 7.56, 2.46, and 3.03% for IgG, IgA, and IgM, respectively. Based on [13, 14], coefficients lower than 10% can be considered as sufficiently precise to allow the use of a single-well for each sample to determine the concentration of IgG, IgA, and IgM. Hence, each plate (24 wells) allowed to analyse simultaneously 21 colostrum samples and three reference sera.

### Near-infrared prediction

Spectra of colostrum were collected using the visible-near reflectance infrared instrument DS2500 (FOSS Analytical A/S, Hillerød, Denmark). The instrument operates between 400 nm and 2499.5 nm, with a 0.5 nm resolution, for a total of 4200 spectral variables. For each sample, 10 mL of thawed colostrum were dispensed after inversion in a slurry cup at room temperature and the instrument automatically averaged 32 sub-spectra collected on eight sample areas by rotating the cup. Spectra with poor quality, i.e., high SD between sub-spectra, were discarded (n = 5). The reference data obtained by RID analysis were paired with spectral variables expressed as log(1/reflectance). The final NIRS dataset comprised 693 samples with spectra and at least one reference value.

The obtained dataset was divided into two groups using the SURVEYSELECT procedure of the SAS software v. 9.4 (SAS Institute Inc., Cary, NC, USA). Random sampling was performed by setting parity order as strata to ensure that all lactations were equally represented in the resulting subsets. In particular, the distribution of records across parities 1, 2, 3, 4, and 5 to 8 was: 28.9, 29.7, 18.4, 12.1, and 10.9%, respectively. For each trait, one subset (28% of the total observations) was used as calibration set, and the second (72% of the total observations) as validation set. The validation set was used neither to train nor to optimize the prediction models but only to evaluate the performance of the final models. Prediction models were developed from the calibration dataset with the WinISI software v. 4.8 (FOSS Analytical A/S, Hillerød, Denmark) using a modified partial least squares algorithm. Different mathematical treatments on spectral data were tested: none; detrend; standard normal variate; standard normal variated and detrend; and multiplicative scatter correction. All the previous treatments were tested in combination with derivative and smoothing parameters as follows: 0,0,1,1; 1,4,4,1; 1,8,8,1; 2,5,5,1; 2,10,10,1, where the first digit is the derivative, the second digit is the gap over which the derivative was calculated, the third digit is the gap for the first smoothing, and the fourth is the gap for the second smoothing. In total, 25 models were developed for each trait. To avoid overfitting, which results in poor validation performances and poor models’ applicability, the number of latent variables (LV) used for building the models was carefully tuned. For this reason, an internal cross-validation in the calibration set was performed using 20 random groups. Within each mathematical treatment, the selected number of LV was the smallest one with a root mean squared error in cross-validation (RMSE_CV_) that was not statistically different from the lowest RMSE_CV_ among all the tested LV [15]. For each mathematical treatment, before the calculation of the final model, three steps of modified partial least squares algorithm were performed. At each step, the dataset was checked for outliers, which were removed before the subsequent iteration. Potential outliers were detected based on a (i) spectral H > 10 (H = D^2^/LV, where D is the Mahalanobis distance of each spectra from the spectral centroid), (ii) T statistics > 3, (where T is the difference between the predicted and reference values estimated by Student’s *t* test), and (iii) X statistics > 10 (an estimation of poorly modelled spectra) [16]. Among all the tested mathematical treatments, we selected the one that resulted in the lowest RMSE_CV_. The developed models were used to predict the concentrations of IgG, IgA, and IgM in the validation set, excluding the predicted samples with a GH > 3.5 and NH > 2.0 [17], which are respectively defined as the H distance from the calibration spectra centroid and the H distance from the most similar (nearest) calibration spectra.

Number of selected LV, coefficient of determination in calibration (R^2^_C_), root mean squared error in calibration (RMSE_C_), coefficient of determination in cross-validation (R^2^_CV_), RMSE_CV_, coefficient of determination in external validation (R^2^_V_), root mean squared error in validation (RMSE_V_), and the relative RMSE_V_, which is defined as the ratio between the average value of reference data in the validation set and the RMSE_V_, have been calculated as fitting statistics.

### Estimation of genetic parameters

A genetic analysis was carried out for each trait using a bivariate animal model in which RID measures and NIRS predictions obtained from the 100% calibration set (194 samples) were included as two different traits:

$$\left[\begin{array}{c}{\mathbf{y_{1}}}\\ {\mathbf{y_{2}}}\end{array}\right]=\left[\begin{array}{cc}{\mathbf{X}}_{\mathbf{1}}& {\mathbf{0}}\\ {\mathbf{0}} & {\mathbf{X}_{2}}\end{array}\right]\left[\begin{array}{c}{\mathbf{b}_{1}}\\ {\mathbf{b}_{2}}\end{array}\right]+\left[\begin{array}{cc}{\mathbf{Z}}_{\mathbf{1}}& {\mathbf{0}}\\ {\mathbf{0}}& {\mathbf{Z}}_{\mathbf{2}}\end{array}\right]\left[\begin{array}{c}{\mathbf{a}}_{\mathbf{1}}\\ {\mathbf{a}_{2}}\end{array}\right]\text{+}\left[\begin{array}{c}{\mathbf{e}}_{1}\\ {\mathbf{e}}_{\mathbf{2}}\end{array}\right],$$

where $\mathbf{y}$ is the vector of phenotypic observations of the dependent variables (measured and predicted trait); $\mathbf{b}$ is the vector of fixed effects (parity, calving season, and herd); $\mathbf{a}$ is the vector of random additive genetic effects; $\mathbf{e}$ is the vector of random residuals; and $\mathbf{X}$ and $\mathbf{Z}$ are incidence matrices relating the corresponding effects to the dependent variable. Expectations ($\mathrm{E}$) of the variables were assumed as $\mathrm{E}(\mathbf{y})=\mathbf{X}\mathbf{b}$, $\mathrm{E}(\mathbf{a})=0$, and $\mathrm{E}(\mathbf{e})=0$, and the variances of random effects were assumed as $\mathrm{var}(\mathbf{a})=\mathbf{A}{\upsigma }_{\mathrm{a}}^{2}$ and $\mathrm{var}(\mathbf{e})=\mathbf{I}{\upsigma }_{\mathrm{e}}^{2}$, where ${\upsigma }_{\mathrm{a}}^{2}$ is the additive genetic variance and ${\upsigma }_{\mathrm{e}}^{2}$ is the residual variance, $\mathbf{A}$ is the pedigree-based relationship matrix, and $\mathbf{I}$ is an identity matrix of order equal to the number of records. The heritability (${\mathrm{h}}^{2}$) of both the reference and predicted traits was calculated as the ratio of ${\upsigma }_{\mathrm{a}}^{2}$ to the sum of ${\upsigma }_{\mathrm{a}}^{2}$ and ${\upsigma }_{\mathrm{e}}^{2}$. Phenotypic (${\mathrm{r}}_{\mathrm{p}}$) and genetic correlations (${\mathrm{r}}_{\mathrm{a}}$) were calculated from the estimated phenotypic (${\mathrm{cov}}_{\mathrm{p1,2}}$) and genetic covariances (${\mathrm{cov}}_{\mathrm{a1,2}}$) as follows:

$${\mathrm{r}}_{\mathrm{p}}=\frac{{\mathrm{cov}}_{\mathrm{p1,2}}}{\sqrt{{\upsigma }_{\mathrm{p}1}^{2} \cdot {\upsigma }_{\mathrm{p}2}^{2}}}\, \mathrm{ and }\,{\mathrm{r}}_{\mathrm{a}}=\frac{{\mathrm{cov}}_{\mathrm{a1,2}}}{\sqrt{{\upsigma }_{\mathrm{a}1}^{2} \cdot {\upsigma }_{\mathrm{a}2}^{2}}},$$

where ${\upsigma }_{\mathrm{p}}^{2}$ denotes the phenotypic variance calculated as the sum of ${\upsigma }_{\mathrm{a}}^{2}$ and ${\upsigma }_{\mathrm{e}}^{2}$, and ${\mathrm{cov}}_{\mathrm{p}}$ is the phenotypic covariance between traits calculated as the sum of the additive genetic and residual covariance. To avoid convergence issues due to the small variance components, the reference and predicted IgA and IgM were multiplied by 100. The software ASReml v4 [18] was used to perform the genetic analyses. The same model was used to estimate the correlations between both NIRS and RID values of IgG, IgA, and IgM concentrations. Univariate analyses were carried out on all the traits to estimate least squares means of the previously described fixed effects.

### Effect of IgG calibration dataset size on (co)variance components

Considering that IgG concentration is the key-trait to determine colostrum quality and is the most important feature for the passive transfer to calf [19, 20], two subsets were extrapolated from the full calibration set (100%), i.e., one containing 80% of the samples (n = 156) and a second one with 60% of the samples (n = 117). The subsets were representative of the variability of herds and parities and were used to develop two prediction equations. The method adopted to develop prediction models from the smaller calibration set was the same as that used for the full calibration set and previously described. The idea was to check for differences in terms of prediction accuracy, heritability of the NIRS-predicted trait, and genetic correlation with the reference IgG concentration when using less reference samples. The linear animal model described above was used to estimate (co)variance components and phenotypic and genetic correlations of reference IgG concentration with its NIRS-predictions with different sizes of calibration sets. In addition, the difference in terms of selection response between reference and NIRS-predicted IgG concentrations was estimated as proposed by [21]. Briefly, the ratio between the correlated (${\mathrm{\Delta G}}_{\mathrm{C}}$, NIRS-predicted IgG) and the direct response ($\mathrm{\Delta G}$, reference IgG) to selection was used to compare the effectiveness of the reference trait ($\mathrm{y}$) and NIRS-predicted trait ($\mathrm{x}$):

$$\frac{{{{\Delta}}\text{G}}_{\text{C}}}{{{\Delta}}\text{G}}= {\text{i}\cdot {\text{r}}}_{\text{g}}\cdot \sqrt{\frac{{\text{h}}_{\text{x} }^{2}\cdot \left[ 4+\left(\text{n}-1\right)\cdot {\text{h}}_{\text{y}}^{2}\right]}{{\text{h}}_{\text{y} }^{2}\cdot \left[ 4+\left(\text{n}-1\right)\cdot {\text{h}}_{\text{x}}^{2}\right]}},$$

where $\mathrm{i}$ is the selection intensity; ${r}_{\mathrm{g}}$ is the genetic correlation between $\mathrm{x}$ and $\mathrm{y}$; $\mathrm{n}$ is the number of daughters per bull with information, which is assumed here to be equal to 70; ${\mathrm{h}}_{\mathrm{x}}^{2}$ is the heritability of trait $\mathrm{x}$ (at 100, 80, or 60% of calibration set); and ${\mathrm{h}}_{\mathrm{y}}^{2}$ is the heritability of trait $\mathrm{y}$.

Finally, to estimate the response to selection using various combinations of NIRS-predicted IgG, IgA, and IgM, different deterministic scenarios with RID IgG concentration as the main breeding goal were simulated [22] by using the parameters obtained above, assuming various criteria on both the sire and dam sides, considering a generation interval of 6 years (sires) and 4 years (dams), and fixing the intensity of selection to 1.76 (i.e. the top 10% of selected individuals).

## Results

### Data overview and prediction accuracy

The descriptive statistics of the analysed samples are in Table 1. The final number of available records ranged from 587 for IgA to 697 for IgG. The concentration of IgG in samples ranged from 0.68 g/L to 216.70 g/L, averaging 91.77 g/L. The concentrations of IgA and IgM averaged 4.80 g/L and 5.07 g/L, respectively, and both exhibited a greater coefficient of variation than that of IgG. Approximately 12% of the samples with information on RID IgG had a suboptimal concentration (< 50 g/L). Moreover, about 18% of the samples had an IgG concentration lower than 60 g/L and 28% of the samples had an IgG concentration lower than 70 g/L.

**Table 1:** Descriptive statistics of colostrum concentration of immunoglobulins determined by radial immunodiffusion

Trait	n	Mean	Standard deviation	Minimum	Maximum
IgG, g/L	697	91.77	36.47	0.68	216.70
IgA, g/L	587	4.80	3.03	0.13	22.14
IgM, g/L	674	5.07	2.44	0.18	14.01

*IgG* immunoglobulins G, *IgA* immunoglobulins A, *IgM* immunoglobulins M

The calibration performances for the analysed traits and the different calibration datasets are in Table 2. The R^2^_C_ of IgG was greater than 0.95 for all the tested calibration sets, i.e., 100, 80, and 60% of the full set. The RMSE_CV_ and R^2^_CV_ were also similar among the models developed for IgG, ranging from 14.22 to 15.93 g/L and from 0.79 to 0.84, respectively. Although different scatter corrections were selected for each of the three models for IgG, the derivative and smoothing parameters were the same for all of them, except IgA.

**Table 2:** Summary of statistics of near-infrared prediction models for colostrum immunoglobulins concentrations

Trait^a^, g/L	n	Mean	SD	Math treatment	LV	RMSE_C_	R^2^_C_	RMSE_CV_	R^2^_CV_	RMSE_V_	R^2^_V_	Relative RMSE_V_
IgG												
100%	189	88.39	36.08	MSC 2,5,5,1	14	6.72	0.97	14.22	0.84	25.21	0.63	0.27
80%	152	91.14	36.28	SNV 2,5,5,1	14	5.26	0.98	14.55	0.84	29.15	0.53	0.32
60%	115	91.81	35.04	MSC 2,5,5,1	16	3.20	0.99	15.93	0.79	29.46	0.49	0.32
IgA												
100%	153	4.25	2.48	MSC 1,4,4,1	4	1.84	0.45	2.00	0.35	2.23	0.40	0.46
IgM												
100%	187	4.71	2.08	DET 2,5,5,1	2	1.58	0.42	1.73	0.31	2.10	0.32	0.41

*SD* standard deviation, *LV* number of latent variables, *RMSE*_*C*_ root mean squared error in calibration, *R*^*2*^_*C*_ coefficient of determination in validation; *RMSE*_*CV*_ root mean squared error in cross-validation, *R*^*2*^_*CV*_ coefficient of determination in cross-validation, *RMSE*_*V*_ root mean squared error in external validation, *R*^*2*^_*V*_ coefficient of determination in external validation, *Relative RMSE*_*V*_ relative root mean squared error in external validation, expressed as the ratio between the RMSE_V_ (this table) and the mean of the reference trait (Table 1).

*IgG* immunoglobulins G, *IgA* immunoglobulins A, *IgM* immunoglobulins M, *MSC* multiplicative scatter correction, *SNV* standard normal variate, *DET* detrend

^a^Predicted from NIRS spectra using 100, 80, or 60% of calibration set

### Genetic parameters of reference and predicted immunoglobulins concentrations

Preliminary univariate analyses were carried out on all the traits to assess the significance of fixed effects (Table 3). For the three Ig, the trend across parities and across seasons of the NIRS-predicted trait resembled the trend of the reference trait and, in all cases, the parity effect was highly significant (*P* < 0.001) in explaining variability of the trait.

**Table 3:** Least squares means (standard error) and significance (*P*) of fixed effects obtained from the univariate analysis

Trait^a^	Parity						Calving season^b^				Herd	
	1	2	3	4	≥ 5	*P (F-value)*	Winter	Spring	Summer	Autumn	*P(F-value)*	*P(F-value)*
IgG, g/L												
Reference	86.29 (4.83)	84.61 (4.73)	105.91 (4.85)	109.31 (5.73)	112.31 (5.65)	< 0.001 (14.17)	93.58 (4.82)	95.50 (5.46)	102.54 (4.66)	106.64 (4.65)	0.007 (4.06)	< 0.001 (3.93)
100%	87.05 (5.38)	83.81 (5.27)	104.95 (5.43)	112.65 (6.39)	102.78 (6.33)	< 0.001 (10.28)	95.11 (5.37)	94.52 (6.10)	100.39 (5.19)	102.97 (5.19)	0.239 (1.41)	< 0.001 (4.76)
80%	84.44 (5.60)	84.91 (5.49)	103.88 (5.64)	111.71 (6.64)	102.98 (6.59)	< 0.001 (9.28)	97.10 (5.59)	93.89 (6.37)	97.19 (5.41)	102.16 (5.39)	0.433 (0.92)	< 0.001 (3.93)
60%	82.52 (5.46)	83.64 (5.34)	100.39 (5.48)	107.29 (6.47)	96.50 (6.39)	< 0.001 (7.58)	94.05 (5.45)	90.49 (6.18)	92.61 (5.26)	99.11 (5.25)	0.312 (1.19)	< 0.001 (3.25)
IgA, g/L												
Reference	3.61 (0.38)	4.79 (0.37)	6.23 (0.38)	6.41 (0.47)	6.53 (0.46)	< 0.001 (21.60)	5.33 (0.40)	5.13 (0.43)	5.62 (0.36)	5.97 (0.36)	< 0.001 (2.10)	< 0.001 (5.10)
100%	4.01 (0.21)	4.42 (0.20)	5.30 (0.21)	5.25 (0.26)	4.90 (0.25)	< 0.001 (14.39)	4.66 (0.22)	4.73 (0.24)	4.52 (0.20)	5.20 (0.20)	< 0.001 (6.46)	< 0.001 (3.67)
IgM, g/L												
Reference	5.05 (0.35)	4.98 (0.34)	6.47 (0.35)	5.82 (0.41)	5.84 (0.41)	< 0.001 (7.10)	5.31 (0.65)	5.25 (0.40)	6.05 (0.34)	5.91 (0.34)	0.024 (3.16)	< 0.001 (5.00)
100%	4.63 (0.18)	4.50 (0.17)	4.97 (0.18)	5.27 (0.21)	4.96 (0.21)	< 0.001 (5.60)	5.18 (0.18)	4.67 (0.20)	4.49 (0.17)	5.12 (0.17)	< 0.001 (9.41)	0.004 (2.61)

^a^Immunoglobulins G (IgG), A (IgA), and M (IgM) determined with reference method and predicted from NIRS spectra using 100, 80, and 60% of calibration set

^b^Defined as: winter (December to February), spring (March to May), summer (June to August), autumn (September to November)

**Fig. 1:**
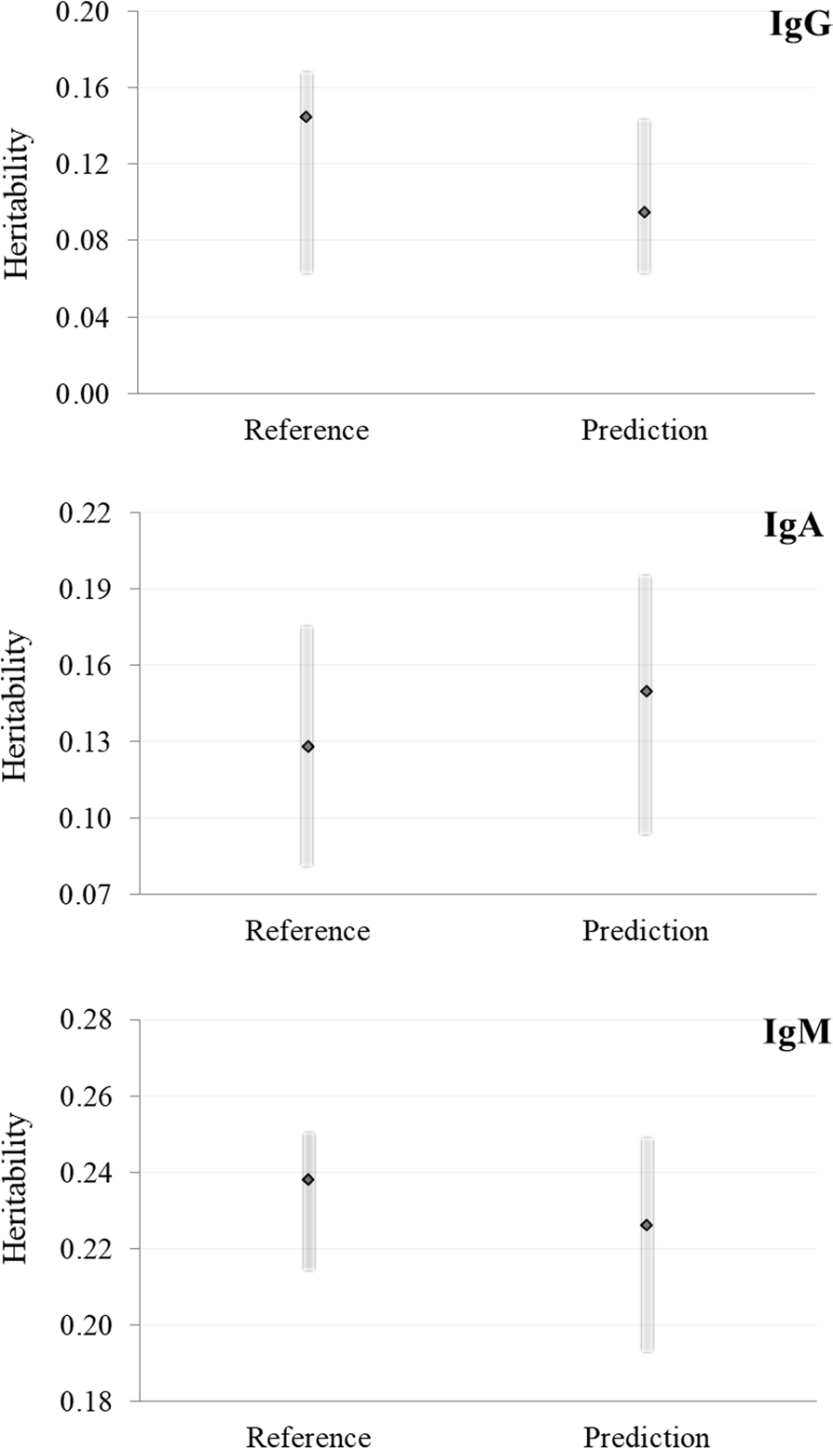
Heritability of reference and infrared-predicted colostrum concentrations of immunoglobulins. The indicator (black diamonds) corresponds to the average of the heritability estimates obtained from the bivariate analyses reported in Table 4, and the grey bar indicates the observed range of heritability estimates. *IgG* immunoglobulins G, g/L; *IgA* immunoglobulins A, g/L; *IgM* immunoglobulins M, g/L

A series of bivariate analyses was used to estimate the genetic covariance, the genetic and residual variances and heritability (Fig. 1). Considering all the bivariate analyses, the heritability of reference IgG, IgA, and IgM concentrations averaged 0.15, 0.13, and 0.24, respectively. Compared to these estimates, the average heritability of NIRS-predicted IgG and IgM concentrations was lower (0.09 and 0.23, respectively), while that of predicted IgA was slightly higher than its reference value (Fig. 1).

The range of heritability estimates was smaller for predicted IgG than for reference IgG concentrations (0.087 vs 0.112; Table 4), while it was larger for predicted IgM than for reference IgM concentrations (0.059 vs 0.033; Table 4). Regarding IgA, the range of the estimated heritability was 0.100 for the reference and 0.105 for NIRS prediction (Table 4).

**Table 4:** Minimum and maximum heritability of colostral immunoglobulins concentrations and their phenotypic (below diagonal) and genetic correlations^a^ (above diagonal)

Trait^b^	Heritability		Reference			Prediction		
	Minimum	Maximum	IgG	IgA	IgM	IgG	IgA	IgM
Reference								
IgG	0.060	0.172	–	0.220 (0.733)	0.401 (0.420)	0.854* (0.314)	0.989 (0.560)	0.738* (0.256)
IgA	0.078	0.178	0.554* (0.036)	–	0.144 (0.556)	Not converged	0.118 (0.784)	0.262 (0.726)
IgM	0.216	0.249	0.603* (0.031)	0.520* (0.037)	–	− 0.050 (0.828)	0.264 (0.470)	0.521 (0.318)
Prediction								
IgG	0.058	0.145	0.767* (0.019)	0.533* (0.036)	0.551* (0.033)	–	0.897 (0.443)	0.761* (0.221)
IgA	0.090	0.195	0.724* (0.024)	0.554* (0.035)	0.567* (0.035)	0.740* (0.022)	–	0.981* (0.174)
IgM	0.195	0.254	0.775* (0.019)	0.569* (0.035)	0.583* (0.032)	0.821* (0.016)	0.778* (0.020)	–

*IgG* immunoglobulins G, *IgA* immunoglobulins A, *IgM* immunoglobulins M

*Significantly different from zero (P < 0.05)

^a^Standard errors of estimates are given in parentheses

^b^Concentrations (g/L) were measured through reference analysis and predicted from colostrum spectra using 100% of the calibration set

The genetic and phenotypic correlations between reference and NIRS-predicted traits are in Table 4. Regarding reference (RID) traits, the magnitude of the phenotypic correlations ranged from 0.520 (IgM and IgA) to 0.603 (IgM and IgG), while the genetic correlations were weaker, with a magnitude ranging from 0.144 (IgA and IgM) to 0.401 (IgG and IgM; Table 4).

The phenotypic correlations between NIRS-predicted IgG, IgA, and IgM concentrations were strong and ranged from 0.740 (between IgA and IgG) to 0.821 (between IgM and IgG), and the genetic correlations were 0.761 (between IgG and IgM), 0.897 (between IgG and IgA), and 0.981 (between IgA and IgM). This did not corroborate the findings for the reference trait, for which the phenotypic correlations between IgG, IgA, and IgM were stronger than their genetic counterparts.

A strong genetic correlation was estimated between reference and predicted IgG concentrations (0.854), while the genetic correlations between reference and predicted IgA and IgM concentrations were weaker (Table 4). Colostrum IgG concentration was the only trait for which the genetic correlation between reference and predicted values was stronger than its phenotypic counterpart (Table 4). The phenotypic associations between reference and predicted IgA and between reference and predicted IgM concentrations were 0.554 and 0.583, respectively. Table 5 summarizes the results of the bivariate analyses carried out for IgG using the different calibration sets (full, 80% and 60%) that were generated in this study.

**Table 5:** Heritability of near infrared-predicted immunoglobulins G concentrations and their correlations with the reference trait

Trait, g/L	Heritability^a^	Correlation^a^	
		Phenotypic	Genetic
Near-infrared prediction			
100% calibration set	0.090 (0.147)	0.767* (0.019)	0.854* (0.314)
80% calibration set	0.102 (0.145)	0.699* (0.024)	0.837* (0.320)
60% calibration set	0.108 (0.139)	0.680* (0.025)	0.846* (0.317)

*Significantly different from zero (P < 0.05)

^a^Standard errors are given in parentheses

**Fig. 2:**
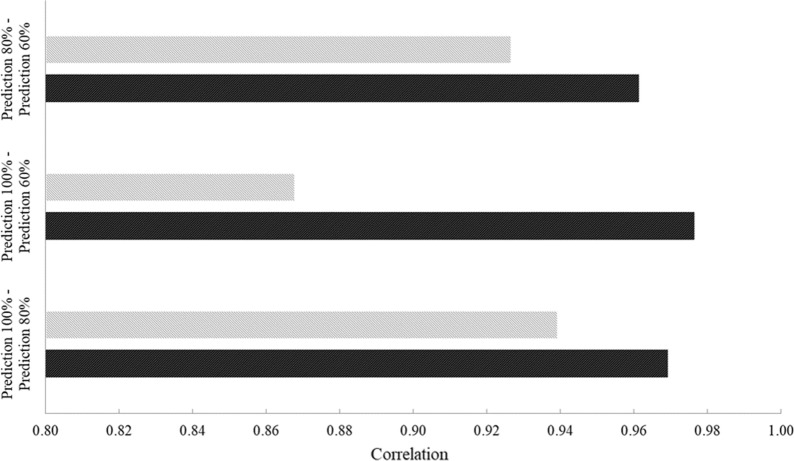
Phenotypic (grey bar) and genetic correlations (black bar) between concentrations of immunoglobulins G (P < 0.05) predicted from NIRS spectra using 100, 80, or 60% of the calibration set

The average heritability of NIRS-predicted IgG concentration was similar for the three calibration sets, i.e. 0.090, 0.102, and 0.108 (Table 5). Both the genetic and phenotypic correlations with the reference IgG concentration were strong in all cases, but the phenotypic correlation decreased slightly when the size of the calibration set decreased (Table 5). The three NIRS-predicted colostrum IgG concentrations (i.e., 100, 80 and 60% of calibration set) were strongly correlated both phenotypically and genetically to each other (Fig. 2) and, in general, the genetic correlations tended to be stronger than their phenotypic counterparts.

Response to selection was calculated for the breeding scenarios in which RID IgG concentration was considered as the breeding goal and various combinations of colostrum traits were included in the selection index (Table 6).

**Table 6:** Response to selection in the breeding goal^a^ under different scenarios

Scenario	Selection criteria	Bulls			Dams				Overall R_y_
		N	R_g_	r_IH_	OP	n	R_g_	r_IH_	
Base	RID IgG	30	9.730	0.730	1	3	6.310	0.480	2.823
Alternative									
I	RID IgG	120	12.030	0.910	1	3	6.310	0.480	3.228
II	NIRS IgG	30	7.230	0.540	1	3	4.290	0.330	2.028
III	NIRS IgG	120	9.700	0.730	1	3	4.290	0.330	2.462
IV	NIRS IgG + IgA + IgM	30	9.370	0.710	1	3	6.436	0.486	2.782
V	NIRS IgG + IgA + IgM	120	11.410	0.860	1	3	6.436	0.486	3.141

*n* number of offspring, *R*_*g*_ response (g/L) per generation, *r*_*IH*_ index accuracy, *OP* own performance, *R*_*y*_ response (g/L) per year, *IgG* immunoglobulins G (g/L), *IgA* immunoglobulins G (g/L), *IgM* immunoglobulins G (g/L), *RID* radial immunodiffusion (direct measure), *NIRS* near-infrared spectroscopy (indirect measure)

^a^Colostrum concentration of immunoglobulins G (g/L) measured through gold standard radial immunodiffusion

The number of offspring assumed per bull was 30, i.e., equal to the minimum number of progeny for officially daughter-proven Italian Holstein bulls. If only RID IgG concentration was used as selection criterion (base scenario), a potential annual genetic gain of + 2.823 g/L could be achievable (Table 6). Moving from 30 to 120 offspring with a phenotypic RID IgG record on the bulls’ side could result in an improved annual genetic gain (2.823 *vs.* 3.228 g/L): + 14.34% compared to the base scenario (Table 6). When only the NIRS-predicted IgG, IgA, and IgM concentrations are used as selection criteria (alternative scenario ‘IV’) to improve the breeding goal (RID IgG), the achievable genetic gain assuming 30 progeny per bull is just 1.46% lower compared to that of the base scenario (Table 6). Again, a four-fold increase in the number of bulls’ progeny with NIRS-predicted IgG, IgA, and IgM concentrations (alternative scenario ‘V’) would potentially translate into a larger genetic gain compared to the base scenario (+ 11.26%; Table 6).

**Fig. 3:**
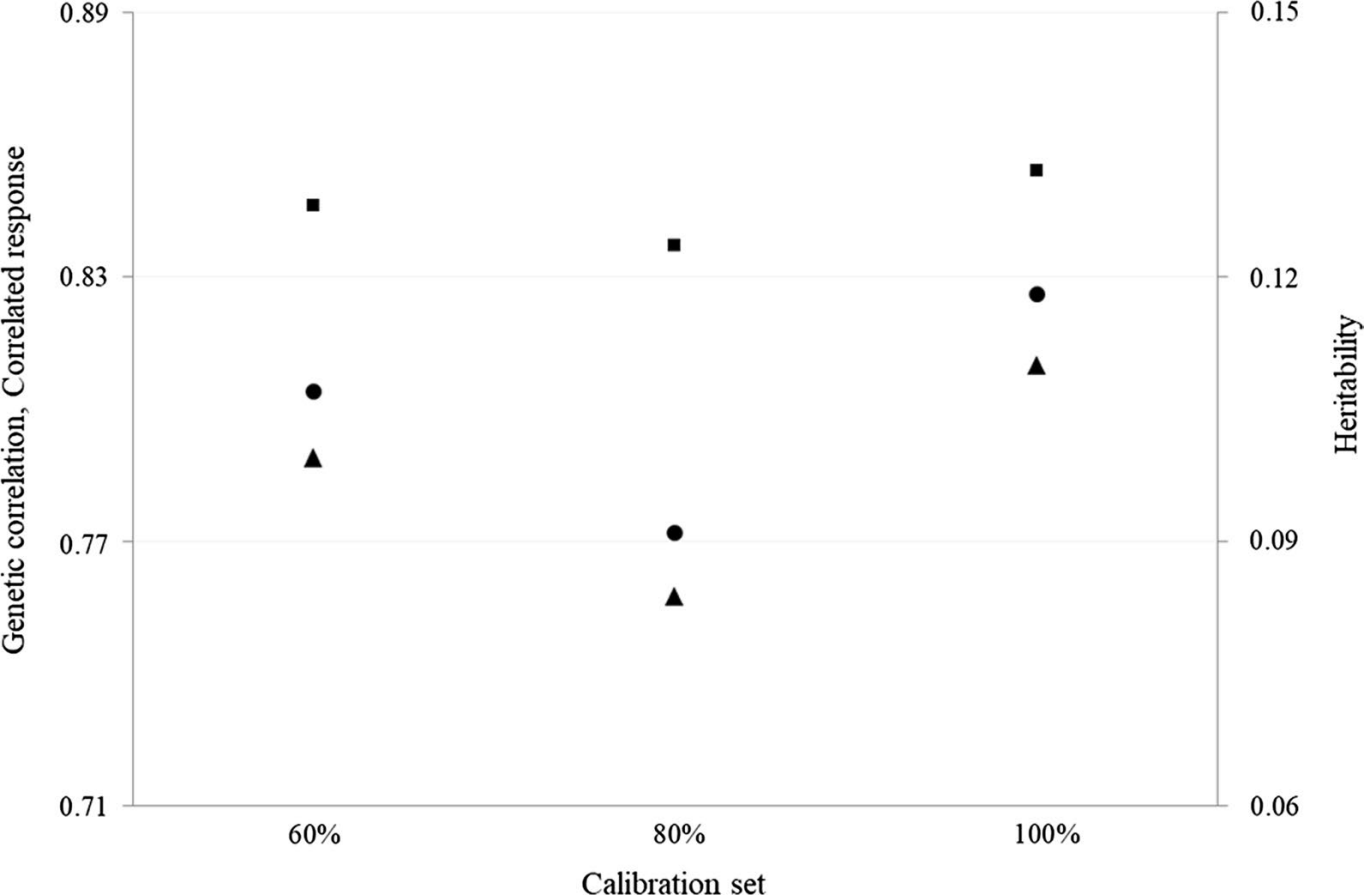
Heritability (black circles), genetic correlation (black squares), and correlated response (black triangles) for colostrum concentrations of immunoglobulins G. Heritability: the average heritability from estimates of bivariate analyses was used for calculations. The correlated response was calculated as the ratio between the indirect (based on infrared-predicted trait) and direct (based on reference trait) response to selection. The colostrum concentrations of immunoglobulins G was predicted from NIRS spectra using 100, 80, and 60% of the calibration set

As shown in Fig. 3, the calculations showed that selecting for NIRS IgG concentration predicted using a calibration set at 100, 80, and 60% resulted in an estimated correlated response of 81, 76, and 79% of the total response that can be achieved with the reference RID IgG, respectively.

## Discussion

The objective of the present study was to predict colostral IgG, IgA, and IgM concentrations in Holstein cows and then to quantify the genetic association between the reference and NIRS-predicted Ig concentrations. The reference methodology used here was the RID assay. Although accurate and repeatable, it is costly, time-consuming, and not feasible for large-scale phenotyping. In this study, NIRS was proposed as an advantageous alternative method for indirect determination of IgG, IgA, and IgM concentrations for breeding and management purposes. NIRS analysis is known to be easy to carry out, rapid, non-destructive, and cost-effective. Moreover, only a small volume of colostrum (10 mL) is necessary for spectra acquisition. The indirect tools available for on-farm Ig prediction are refractometers. Colostral BRIX generally assesses the total solids content, which is related to IgG concentration, i.e., the target trait for quality evaluation. Refractometers can be used on-site by farmers without requiring expert personnel, specific training, and expensive equipment. Nevertheless, the correlation between colostrum BRIX refractive index and IgG concentration can vary depending on the bovine milk samples and often it is not strong [1, 4, 5, 20]. For these reasons, NIRS is a promising method to provide accurate information not only on colostral IgG concentration, but also on several other traits of interest, such as the concentration of total protein, fat, fatty acids, amino acids, minerals, vitamins, growth factors, and oligosaccharides [8, 9, 23].

From a practical point of view, the collection of colostrum aliquots for NIRS analysis requires limited extra labour at the farm level, since farmers are requested to store and label the tubes intended for infrared analysis. However, less than 10 mL of colostrum are needed for spectra collection through the FOSS DS2500 and most dairy farmers already collect and store colostrum to resupply the farm colostrum bank [24]. In addition, the colostrum sample needs to be only collected once per cow in a given lactation and can be stored at a standard freezer operating temperature (− 20 °C) until the day of analysis. Practically, samples of colostrum that are suitable for NIRS analysis can be sent to a laboratory that is equipped with a NIR instrument during the monthly visit within the official milk recording programme, which also allows its evaluation and the storage of compositional information for breeding purposes.

The average IgG concentration obtained in our study agreed with findings on IgG1 concentrations reported for 77 Holstein cows reared on an experimental French farm [25]. It is worth noting that bovine colostrum contains two forms of IgG, namely IgG1 and IgG2, with concentrations representing 80 and 20% of the total IgG, respectively [26]. Le Cozler et al. [25] reported an average RID IgG1 concentration of 54.1 g/L in composite colostrum with a minimum lower than 10 g/L and a maximum of 110.8 g/L. In the same study, significant differences were observed in terms of IgG1 concentration between the hind (56.2 g/L) and front quarters (53.1 g/L). In our study, the RID assay was able to detect the overall IgG content, without discriminating between IgG1 and IgG2. Our results in Table 1 are consistent with those reported by Rivero et al. [9] on Chilean Holsteins with an average RID IgG concentration of 93.3 g/L; moreover, the average concentrations of IgA and IgM at first milking observed in Holstein cows in Brasil [27] were similar to those found in our study (Table 1). As suggested by [28], differences in Ig concentrations could be attributable to the method used for their quantification and to the time of sampling. In fact, the concentration of antibodies is known to rapidly decrease in cattle during the first hours after calving and it can vary if the calf is left with the dam and allowed to suckle. Differences with other studies are small and may be due to sampling protocol and reference analysis. In particular, it is important to highlight that the samples used in our study represent the first colostrum, because they were collected during a short period of time after parturition (6 h, at the latest). In fact, according to the protocol adopted, calves were not allowed to suckle as they were immediately separated from the dam after birth.

The production of low-quality first colostrum, i.e., colostrum with an IgG concentration lower than 50 g/L, is quite frequent and it concerned 12% of the cows sampled in our study; this phenomenon impairs the passive transfer of immunity from the dam to the calf and has a negative impact on the calves’ health and performance [24].

### Prediction accuracy

Infrared prediction models used in this study were built to generate potential traits that evaluate colostrum quality at the population level. The convergence of genetic analyses on such traits requires a large number of samples with NIRS-predicted and reference values. For this reason, most samples were included in the validation set rather than in the calibration set. To the best of our knowledge, few studies have reported NIRS prediction models to determine colostrum compounds. By using 90 samples in the calibration set, some authors have reported good NIRS prediction performances for major components of the bovine colostrum, such as content in total solids, fat, solid non fat (SNF), lactose, and protein [8]. Nevertheless, only cross-validation was performed. As reported here, there could be a large difference between cross-validation and external validation fitting statistics (Table 2). In a previous research [9], NIRS prediction models for colostrum IgG content were developed starting from 157 samples, using a protocol similar to that used in our study. The authors [9] reported R^2^_CV_ and RSME_CV_ of 0.94 and 9.03 g/L, respectively, and the reported fitting statistics were better than those obtained in our study, but again no external validation was performed. Our findings highlighted the suitability of NIRS-predicted Ig for genetic investigations and for monitoring colostrum quality. Accordingly, the development of more robust prediction models, including a larger number of samples in the calibration dataset and using more complex algorithms, could definitely be an improvement.

In this study, RMSE_V_ increased as the number of samples included in the calibration dataset decreased, which could indicate overfitting of the models, even if cross-validation was performed for the optimization of the number of LV, or an insufficient sample size in calibration. Moreover, the selected mathematical treatment applied to the 80% calibration set differed to that selected for the full and for the 60% calibration sets. Finally, although the three sets were similar in terms of mean and variability, the samples in the reduced calibration sets were selected randomly, which might have excluded (or included) some slightly leveraged spectral data.

### Genetic variability of colostrum immunoglobulins and practical implications

The occurrence of diseases and metabolic disorders in dairy cattle are a major source of economic losses for farmers. Therefore, the ability to genetically improve animal health through selective breeding is receiving growing attention worldwide. The presence of exploitable genetic variation has been demonstrated for a series of animal diseases, such as clinical mastitis, ketosis, lameness, metritis, displaced abomasum, and milk fever [29–31]. The implementation of breeding schemes to genetically improve the health of livestock requires a national recording scheme in which diseases and metabolic disorders are systematically registered in a standardized manner. Scandinavian countries were the first to develop recording systems for health traits in cattle in the 1970s and currently, udder, and claw health are major components in their national selection indexes. In these countries, evident and touchable benefits have been reported in terms of reducing disease incidence [32]. Quantification of the additive genetic variance of traits related to the cow or calf immune system, such as antibody concentration, has been carried out by several authors in different biological fluids, i.e., serum [33–36], mature milk [36–38], and colostrum [35]. It has been demonstrated that the concentration of Ig in mature milk is very low [26, 39], which impairs the accurate determination of such traits through individual milk spectra officially collected across the lactation. To the best of our knowledge, no studies have evaluated the correlations between colostral and milk IgG concentrations or investigated the feasibility of infrared spectroscopy measures for predicting milk IgG concentration. If predicting colostral IgG concentration from milk spectra at the first test-day (first month of lactation) is feasible and accurate, it could be a potential strategy to facilitate and promote the collection of data on colostrum quality. Compared to different strategies, IgG can only be predicted with a low coefficient of determination (0.56) based on information collected in the previous lactation, e.g., milk, fat and protein yield, dry period length and fertility parameters in multiparous cows [40]. Investigations have been carried out to determine the gross composition of colostrum (fat, protein and lactose concentration), yield (kg) and content of energy and total solids, the latter being quantified with a BRIX refractometer and reported to be associated with solids concentration, including IgG [19, 20]. The estimated heritability of the BRIX refractive index in Greek Holstein was 0.27 (0.09) [20], which is close to our value for RID IgG concentration and it is genetically correlated with protein content of colostrum (0.97 ± 0.03) [20]. The estimated heritability for RID IgG concentration (0.28 ± 0.14) in Charolais cattle [35] was slightly lower than our value for RID IgG. Possible explanations are related to differences in terms of sampling protocol, statistical model, and thus phenotypic and genetic variation of the trait. A low genetic correlation (0.12 ± 0.65) between RID-measured IgG and ELISA-measured IgG concentrations was reported by the same authors [35]. To our knowledge, this is the only paper reporting estimates of heritability for IgA (0.05) and IgM concentrations (0.22) in the colostrum. It is worth noting that the standard errors of the heritability estimates were large and similar to those reported in other recent papers on cow colostrum traits, where overall standard errors of genetic correlations ranged from 0.03 to 0.73 [20, 35]. In fact, some of the correlations shown in Table 4 were not significantly different from zero, which is likely due to the small sample size that resulted in large standard errors. More work is needed to improve the data and reduce the standard error of the genetic parameters, including the genetic correlations between the three Ig fractions (RID and NIRS).

A potential strategy to indirectly improve health and welfare in calves is to estimate animal genetic merit for colostrum quality. To achieve this goal, large-scale phenotyping is fundamental, yet the resources required to accurately quantify the concentration of antibodies in the colostrum might hamper their implementation in breeding schemes. However, our findings demonstrate that NIRS-predicted Ig concentrations are genetically correlated with their reference counterparts and show that the trends across parities and calving season are similar to those observed for the reference trait (Table 3). This is particularly true for the IgG which are the most abundant immunoglobulins in the colostrum and are reported as the most relevant trait to define colostrum quality.

In the alternative scenario ‘I’ (Table 6), the number of offspring per bull with RID IgG moved from 30 to 120, resulting in augmented cost for RID analysis but in greater genetic gain (+ 14.34%) compared to the base scenario. The prediction models developed here could allow the generation of NIRS-predicted IgG, IgA, and IgM concentrations at a negligible cost and on a large-scale. Yet, selection in Italian Holstein is currently mostly genomic and the goal in this case would be to build up a reference population with phenotype(s) of interest available rather than to increase the number of progeny per bull. Moreover, using NIRS predictions instead of RID traits allows to reduce the costs of phenotyping and to reach a comparable response to selection. Finally, the genetic correlations and the correlated responses calculated for IgG predicted from different calibration sets further support the use of NIRS-predicted IgG as a proxy for breeding purposes. In fact, selecting for NIRS IgG concentration that is predicted by using a calibration set at 100, 80, and 60% allowed us to obtain a similar correlated response, i.e. equal to 81, 76, and 79% of the total response potentially achievable with the reference RID IgG concentration, respectively (Fig. 3). This means that the loss in accuracy seems to be limited.

Studies that have investigated the association between colostral IgG level and subsequent lactation performance in female calves are not recent. As an example, calves fed four litres of high-quality colostrum showed significantly greater milk yield in the first two lactations compared to those fed two litres in a study published in 2005 [41]. Furthermore, DeNise et al. [42] in 1989 observed that, for each additional unit (g) of serum IgG in female calves between 24 and 48 h of age, there was an increase in both milk yield (+ 8.5 kg) and fat yield (+ 0.24 kg) in the first lactation. In brief, calves with a low plasma IgG level (< 10 g/L) at 24 h of age are at major risk of failure of passive transfer and are more susceptible to disease; farmers should aim at reaching an IgG blood concentration higher than 25 g/L in calves at 24 h of age [24]. These references support that a positive association exists between the calves’ survival, health, and performance and the IgG concentration of colostrum, even for colostral IgG concentrations greater than 50 g/L. Thus, the greater is the IgG concentration, the better is the quality of colostrum intended for feeding new-born calves, particularly in the first six hours of life. In our study, 12% of the cows produced colostrum of insufficient quality, i.e., with a RID IgG concentration lower than 50 g/L. The concentration in IgG is essential for an optimal and successful passive transfer of immunity; in fact, colostrum intake in calves may be suboptimal in the first hours after birth, e.g., due to poor management practices or low inappetence, thus it is important to maximise IgG (g) per L. Failure of passive transfer is a condition that has negative consequences in the short, medium and long term in young animals and is reported to be one of the major causes of death in early life [24]. A proper postnatal colostrum intake coupled with feeding administration strategies positively affect growth rates, health, fertility, and productive performance, and promote the raising of robust future heifers [43]. According to studies carried out in the US and Scotland, around 15% of dairy calves experience failure of passive transfer [24, 44], corroborating the number of suboptimal-quality samples (12%) observed in this study.

It is reasonable to assume that, due to physiological limitations, IgG concentration can be improved through breeding only up to a certain level in dairy cattle. In addition, it is likely that colostrum yield (kg) and quality are inversely correlated. In this view, ideally, both aspects should be combined to define an efficient breeding strategy, e.g., by considering the total amount of IgG yielded (g) by a cow in the first 6 h after calving.

A further complication is that the calf breeding value must also be accounted for in order to plan robust strategies that are aimed at reducing the incidence of failure of passive transfer. Finally, as it can occur on farms using an internal colostrum bank, the cow that produces the administered colostrum may not be genetically linked to the calf.

## Conclusions

In this study, we used available samples of first colostrum to evaluate the ability of NIRS to predict the concentration of Ig and assess the genetic correlations between the reference and the NIRS-predicted trait. In this view, NIRS could be a cost-effective and rapid method to collect phenotypes on colostrum quality for breeding purposes. Both reference and NIRS-predicted IgG, IgA, and IgM concentrations were heritable and showed genetic variation. In addition, NIRS-predicted IgG, IgA, and IgM concentrations were genetically correlated with their reference counterparts, particularly in the case of IgG, which is in charge of the passive transfer of immunity from the dam to the calf. Simulations were carried out to estimate genetic gain in terms of colostrum quality by using estimated genetic parameters. Overall, the simulated scenarios indicated that NIRS-predicted IgG, IgA, and IgM concentrations could be exploited in the future for selective breeding to improve the quality of first colostrum secreted during the *post-partum* period by the mammary gland of Holstein cows. Nevertheless, given the large standard errors of the estimated genetic parameters, further phenotyping is on-going to improve the size of the dataset and explore other genetic and genomic aspects. Among the perspectives, the ability of infrared spectroscopy to predict colostral IgG concentration from milk spectra at the first test-day (first month of lactation) could be evaluated, since it would definitely facilitate and speed-up the collection of colostrum quality data.

## Data Availability

The datasets generated and/or analysed during the current study are not publicly available but are available from the corresponding author on reasonable request.
